# Antarctic benthic diatoms after 10 months of dark exposure: consequences for photosynthesis and cellular integrity

**DOI:** 10.3389/fpls.2024.1326375

**Published:** 2024-03-22

**Authors:** Jacob Handy, Desirée Juchem, Qian Wang, Katherina Schimani, Oliver Skibbe, Jonas Zimmermann, Ulf Karsten, Klaus Herburger

**Affiliations:** ^1^ Cell Biology of Phototrophic Marine Organisms, Institute of Biological Sciences, University of Rostock, Rostock, Germany; ^2^ Applied Ecology and Phycology, Institute of Biological Sciences, University of Rostock, Rostock, Germany; ^3^ Botanischer Garten und Botanisches Museum Berlin, Freie Universität Berlin, Berlin, Germany

**Keywords:** Antarctica, dark adaptation, diatoms, photosynthesis, polar night, plastoglobules

## Abstract

Antarctic algae are exposed to prolonged periods of extreme darkness due to polar night, and coverage by ice and snow can extend such dark conditions to up to 10 months. A major group of microalgae in benthic habitats of Antarctica are diatoms, which are key primary producers in these regions. However, the effects of extremely prolonged dark exposure on their photosynthesis, cellular ultrastructure, and cell integrity remain unknown. Here we show that five strains of Antarctic benthic diatoms exhibit an active photosynthetic apparatus despite 10 months of dark-exposure. This was shown by a steady effective quantum yield of photosystem II (Y[II]) upon light exposure for up to 2.5 months, suggesting that Antarctic diatoms do not rely on metabolically inactive resting cells to survive prolonged darkness. While limnic strains performed better than their marine counterparts, Y(II) recovery to values commonly observed in diatoms occurred after 4-5 months of light exposure in all strains, suggesting long recovering times. Dark exposure for 10 months dramatically reduced the chloroplast ultrastructure, thylakoid stacking, and led to a higher proportion of cells with compromised membranes than in light-adapted cells. However, photosynthetic oxygen production was readily measurable after darkness and strong photoinhibition only occurred at high light levels (>800 µmol photons m^-2^ s^-1^). Our data suggest that Antarctic benthic diatoms are well adapted to long dark periods. However, prolonged darkness for several months followed by only few months of light and another dark period may prevent them to regain their full photosynthetic potential due to long recovery times, which might compromise long-term population survival.

## 1 Introduction

Studying organisms with adaptation capacities to extreme environments is crucial, especially given the current climate change scenarios that predict dramatic alterations of abiotic conditions in evolutionary short periods in many environments worldwide (Turner et al., 2020). This is particularly relevant in vulnerable regions like Antarctica, where organisms must adapt to prolonged periods of darkness or continuous light and cope with very low temperatures (Cottier and Potter, 2020). Antarctica is characterized by extensive ice and snow cover, and significant light variations, including long periods of complete darkness. The lack of light during the three to 6 months of polar night is a major challenge for primary producers in these regions (McMinn et al., 2010; Cottier and Potter, 2020). Sea ice can extend the dark period, reducing sunlight to only 2% of normal levels in some areas for up to 10 months (Karsten et al., 2019a). Together with overlying snow cover, 99.9% of the remaining surface irradiance are cut out (Petrich and Eicken, 1998), frequently yielding light levels of less than 5 μmol photons m^−2^ s^−1^ beneath the sea ice in summer (McMinn et al., 1999). In parts of Antarctica, such as the Weddell Sea, large areas of unbroken fast ice can persist for two or more seasons, resulting in lack of light for several months (Peters and Thomas, 1996). Thus, polar photosynthetic organisms must adapt to darkness/very low light conditions for periods that can exceed the polar night by months, but also cope with high light in summer, displaying flexibility in their photosynthetic efficiency (Longhi et al., 2003; Gómez et al., 2009; Zacher et al., 2009).

An important group of organisms in this context are diatoms (Bacillariophyta); these microalgae dominate well-mixed water columns across all oceans, benthic algal communities of shallow-water soft bottoms and rock biofilms (Lacour et al., 2019). Diatoms are responsible for 20-25% of global and up to 50% of marine primary production, producing more oxygen than all rainforests combined (Field et al., 1998). At depths of 30 meters, diatoms serve as the main food source for benthic suspension/deposit feeders, thus occupying a significant position in polar coastal food webs (Kowalke, 1998; Glud et al., 2009). Moreover, as primary producers, benthic diatoms and their planktic counterparts play a crucial role in global carbon dioxide fixation, binding approximately 45% of marine CO_2_ (Mann, 1999). The physiological state of polar diatoms surviving in darkness and their underlying cellular/physiological processes remain largely unstudied. This is even though diatom long-term dark survival might have been a key adaptive trait to survive the Cretaceous mass extinction events, where diatoms experienced a relatively slight generic loss compared to other planktonic organisms such as coccolithophores (Ribeiro et al., 2011). Thus, the implications of dark survival in diatoms may therefore be far reaching, and may be a contributing factor to the dominance of diatoms within algal communities of polar regions and elsewhere. Various mechanisms have been observed in a few diatoms to adapt to the polar night (Reeves et al., 2011). This includes a reduced metabolic activity (Palmisano and Sullivan, 1982; Peters and Thomas, 1996), maintenance of membrane integrity (Karsten et al., 2019a, b), utilization of stored energy reserves like chrysolaminarin or lipids (Karsten et al., 2012; Schaub et al., 2017; Juchem et al., 2023), formation of resting stages (McQuoid and Hobson, 1996; Durbin, 1978), phototaxis and/or switching to a mixotrophic lifestyle (Hellebust and Lewin, 1977; Tuchman et al., 2006). A recent study on five Antarctic benthic diatoms revealed their reliance on storage lipids during a 3-month dark exposure experiment (Juchem et al., 2023). During this time, a decline of photosynthetic performance was found for two species, while all five displayed some chloroplast degradation. However, the effects of extended dark exposure on photosynthesis, cellular ultrastructure, and cell integrity in diatoms remain largely unknown. This is a problem as it prevents us from understanding the long-term population survival of these key photosynthetic organisms. For example, whether they can easily restore their populations from a long-lasting dark-adapted state relying on autophagy and/or lipolysis, require input of viable cells from neighboring areas with less extreme light conditions or from dormant resting spores (cysts).

Here, we explored the consequences of prolonged dark adaptation (10 months) on the photo-physiological performance, cellular structure, and survivability of five diatom strains isolated from Antarctic benthic habitats. We hypothesized that when exposed to light, these algae can rapidly regain photosynthetic activity, despite prolonged dark exposure, which would suggest a swift restoration of high metabolic activity.

## 2 Materials and methods

### 2.1 Algal origin and cultivation

Five benthic diatoms cultures established at the Botanic Garden and Botanical Museum Berlin (Germany) were maintained in the in-house algal culture collection at the University of Rostock. Algal samples were collected during a field trip to Antarctica in 2020. Diatom strains isolated from these samples were taxonomically characterized as described elsewhere (Prelle et al., 2022; Juchem et al., 2023). The four different sampling sites, three of which marine and one limnic, are shown in **Supplementary Figure S1** and described in **Supplementary Table S1**. Information on the isolation and establishment of unialgal cultures can be found in Juchem et al. (2023). The diatoms were cultured in flasks containing sterile-filtered Baltic Sea water enriched with Guillard’s f/2 medium (Guillard and Ryther, 1962) and 0.6 mM metasilicate (Na_2_SiO_3_·5 H_2_O). Salinity was set to 33 S_A_ for the marine cultures (*Navicula criophiliforma*, *Chamaepinnularia gerlachei*, *Melosira* sp.) by adding artificial sea salt (hw-Marinemix® professional, Wiegandt GmbH, Germany) or to 1 S_A_ for limnic cultures (*Planothidium wetzelii* D300_015 + 025) by dilution with deionized water. Nutrients were replenished by changing the media regularly. Algae were maintained at 8° C and 15 μmol photons m^−2^ s^−1^ under a 16/8-h light/dark cycle set in a culture chamber. For light exposure following dark adaptation experiments, algae were kept at constant 15 μmol photons m^−2^ s^−1^ at 5° C for up to 5 months.

### 2.2 Experimental design for dark incubation and photosynthesis measurements

Algal flasks were incubated under dark conditions at 5° C for 10 months in a culture chamber. To assess the photosynthetic performance of dark-adapted diatoms, 150 µl of cell suspension was transferred to 96-well microplates in a darkened room using a weak red-light source (PPFD = ~1 µmol m^-2^s^-1^). This was done with two independent replicates each consisting of 3 technical replicates. To ensure comparable biomass in wells, cells were concentrated by filtration. Plates were placed on a cooling block set to the dark incubation temperature of 5° C or a cool metal shelf to prevent temperature stress. Next, the effective quantum yield of photosystem II (Y[II]) of algae was measured, which describes the efficiency of energy transfer from the antenna to photosystem II and is used as a proxy for the overall photosynthetic performance of algae and plants (Herburger et al., 2015). The Y(II) was determined with a pulse amplitude modulation (PAM) fluorimeter (PAM-2500, Heinz Walz GmbH, Effeltrich, Germany). The PAM probe was adjusted ~1 mm above wells containing algae and measurements were performed through the well lid, avoiding disturbance of algae or evaporation of media due to opening. The Y(II) was measured every 15 min for the first 300 min after 10 months of dark adaptation, followed by ~daily measurements for 2.5 months, and after 4 and 5 months. As the Y(II) values did not differ significantly in all five strains during the first 300 min after dark exposure, they were averaged and expressed as day one of light exposure. Between these measurements taking up to 5 months, algae were kept in a culture chamber at 5° C and constant light (15 µmol photons m^-2^ s^-1^). We have chosen these relatively low light intensities to simulate a continued light limitation as it can occur during, for example, snow/ice coverage and to allow for comparability with an earlier study one the five strains investigated (Juchem et al., 2023).

### 2.3 Photosynthetic oxygen production and respiratory consumption

The photosynthetic oxygen production and respiratory consumption was measured in algal cultures that have been dark-adapted for 10 months and measured again in cultures after 2.5 months of light exposure (15 µmol photons m^-2^ s^-1^, 5° C). Photosynthesis-irradiance (PI) curves were recorded at 11 increasing light levels (0~1600 μmol photons m^-2^ s^-1^), each light level was maintained for 30 min (n=4). Algae were enriched with 2 mM NaHCO_3_ to avoid carbon deficiency and transferred to measuring chambers placed on magnetic stirrers. Light was generated by LEDs (LUXEON Rebel1 LXML-PWN1-0100, neutral-white, Philips, Amsterdam, Netherlands). Chambers were connected to an Oxy 4-mini meter (Presens Precision Sensing GmbH, Regensburg, Germany), and data were recorded using the software Presens OXY4v2_30. Temperatures in the chambers were maintained at 5° C using a refrigerated circulator pump system. More details for this experimental setup can be found elsewhere (Prelle et al., 2019). After O_2_ measurements, algae were transferred onto Whatman GF/6 glass fiber filters, chlorophyll *a* was extracted with 96% EtOH (v/v) and quantified spectrophotometrically (Helcom, 2019). Oxygen values were expressed as µmol O_2_ mg^-1^ chlorophyll a h^-1^ and PI curve data points fitted using the model of Walsby (1997) to derive photosynthetic parameters. The risk of overestimating diatom respiration due to the presence of bacteria in cultures was considered low, because removing diatoms from culture medium by sedimentation, followed by measuring O_2_ consumption as described above produced only negligible consumption values.

### 2.4 Cell integrity and cell number dark- versus light-adapted

Cell integrity of 10 months dark-adapted samples was compared with samples exposed to 15 µmol photons m^-2^ s^-1^ (5° C) for 8 days and for 2.5 months by using SYTOX Green staining (n=2), which yields green fluorescence after entering cells with compromised membranes. Cells were harvested, stained with 0.5 µM SYTOX Green (Catalog no. S7020, Thermo Fisher Scientific, Waltham, Massachusetts, USA) in culture medium for 5-10 min in darkness. Stained and unstained cells were counted using an epifluorescence microscope (BX-51, Olympus/Evident, Hamburg, Germany) equipped with a GFP filter set. At least 400 unstained and stained (compromised) cells were counted per group.

Algal growth (cell number) was determined in samples preserved with 2.5% glutaraldehyde, which were taken after 6, 8 and 10 months of darkness. In a second experiments, cells were taken at the end of the 10 months dark period, after 8 days of light exposure and after 2.5 months of light exposure (15 µmol photons m^-2^ s^-1^, 5° C; n=6). Cells were counted in sterile channel slides (Catalog no. 80601, ibidi, Gräfeling, Germany; 6 channels/slide: channel volume 30 µl). At least 500 cells per sample were counted and expressed as cell number per ml.

### 2.5 Cellular ultrastructure dark- versus light-adapted

To assess ultrastructural changes occurring after prolonged darkness, 10 months dark-adapted algal cells were compared with samples used for Y(II) measurements, which were exposed to 15 µmol photons m^-2^ s^-1^ at 5° C for 2.5 months. Samples were prepared for transmission electron microscopy (TEM) as described previously (Herburger et al., 2015). Briefly, cells were fixed with 2.5% glutaraldehyde in 50 mM cacodylic acid buffer (pH 6.5) for 4 h and postfixed in 1% OsO_4_ in 50 mM cacodylic acid buffer for 22 h. Fixed cells were dehydrated using a gradient of increasing ethanol, embedded in modified low viscosity Spurr resin and sectioned using a Reichert Ultracut S microtome. Ultrathin sections on grids were counterstained with uranyl acetate, lead citrate and examined with a transmission electron microscope (Zeiss EM902; 80 kV) equipped with a 1x2k FT-CCD camera.

### 2.6 Quantifying lipid droplets in cells

The volume of lipid droplets was quantified using Nile red staining (Alemán-Nava et al., 2016). Cells were stained in PBS containing 0.05% nile red crystals (suspended in DMSO) for 10 min, followed by mounting in PBS. Nile red fluorescence was visualized using a Keyence BX-800 digital microscope equipped with a TRITC filter set. Nile red fluorescence images were merged with corresponding bright field images. The volume of lipid droplets and cells was measured using Fiji (ImageJ; Schindelin et al., 2012). Cell volumes were calculated with the assumption of ellipsoid shapes or calculated using two hemispheres and a cylinder (*Melosira* sp.) as done before (Juchem et al., 2023).

## 3 Results

### 3.1 Photosynthetic performance – dark- versus light-adapted

**Figure 1:**
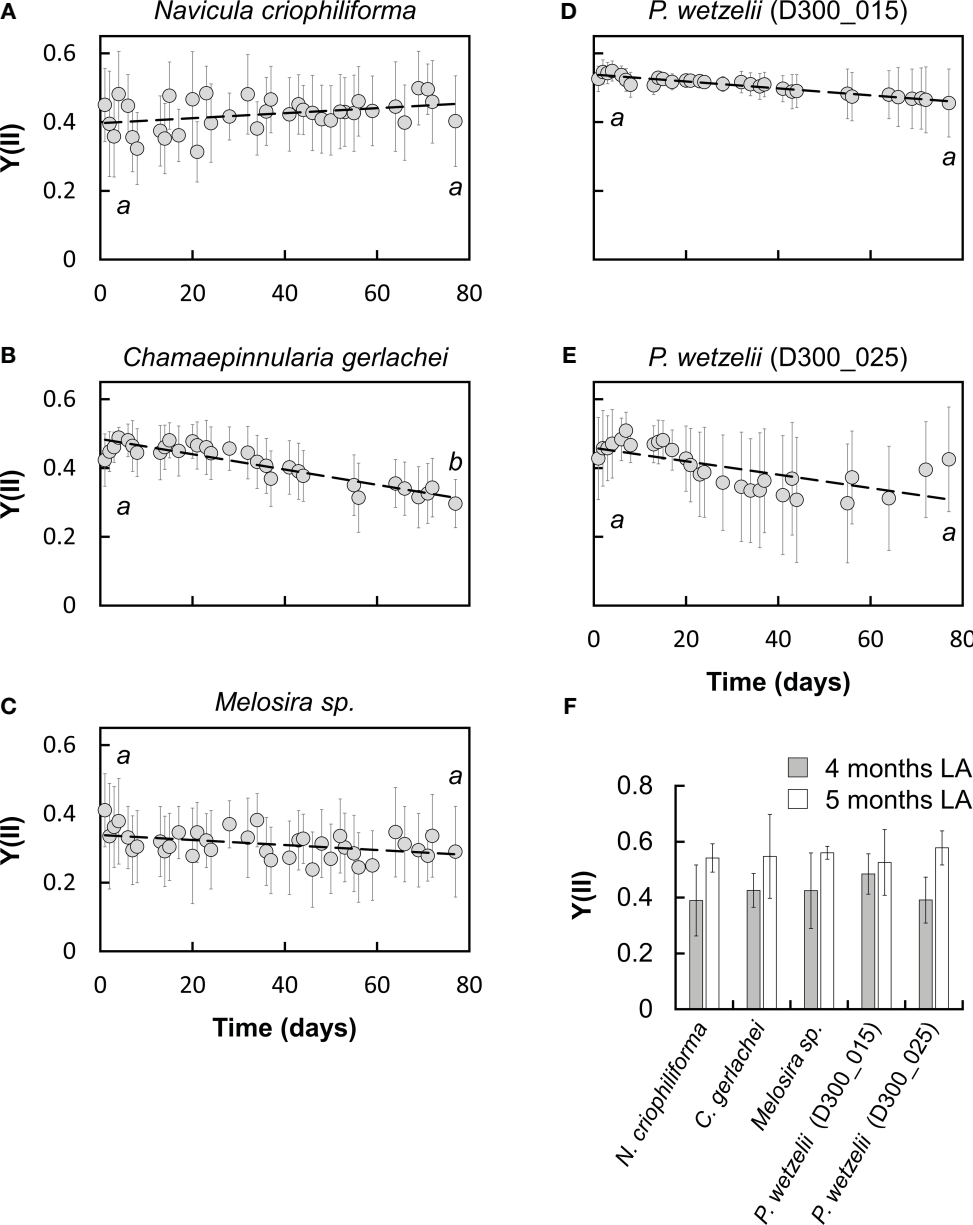
Monitoring the effective quantum yield of PSII (Y[II]) over 2.5 months in five Antarctic benthic diatoms **(A-E)** at 15 µmol photons m^-2^ s^-1^ (n=6± SD: **A**, **C**; n=9± SD: **B**, **D-F**). Samples were taken from cultures after 10 months of dark adaptation. Significances between day one and day 77 of light exposure are indicated by letters and were determined using a student’s t test (p<0.05). **(F)** Y[II] after 4 and 5 months at 15 µmol photons m^-2^ s^-1^.

**Figure 2:**
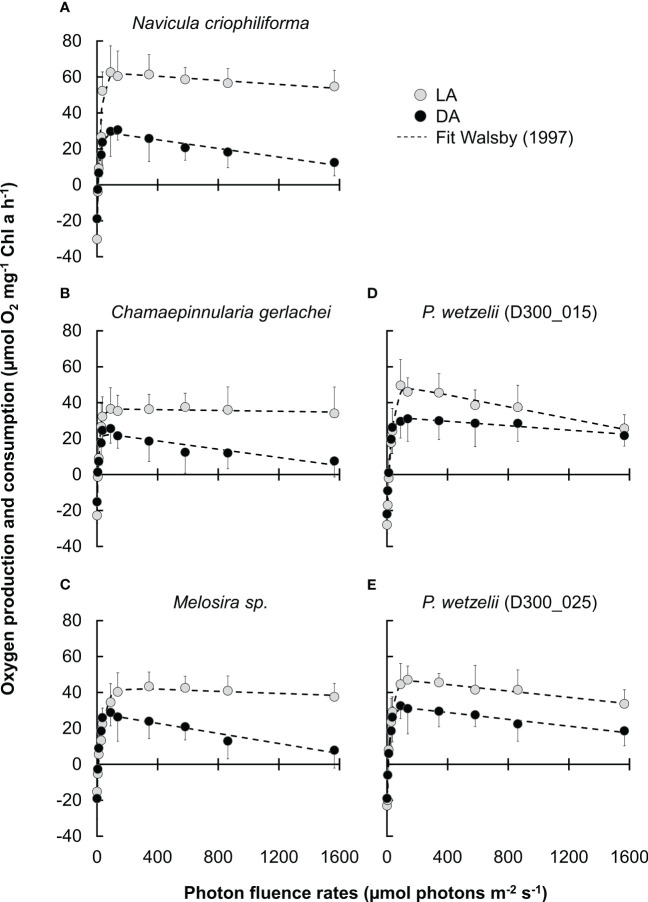
Photosynthetic oxygen production and respiratory consumption in response to increasing PPFD up to ~1600 μmol photons m^−2^ s^−1^ (PI curves, n=4 ± SD). **(A-E)** five Antarctic benthic diatoms exposed to 10 months of darkness (DA), followed by light adaptation at 15 µmol photons m^-2^ s^-1^ for 2.5 months (LA). Data points were fitted according to Walsby (1997).

Despite prolonged dark exposure for 10 months, all five strains showed an appreciable photosynthetic performance during the first day of light exposure (**Figures 1**, **2**). However, the Y(II) differed among strains on day one (**Figure 1**). Limnic *Planothidium wetzelii* (D300_015) consistently produced the highest Y(II) values (~0.5) (**Figure 1D**). In contrast, marine *N. criophiliforma*, *C. gerlachei* and *Melosira* sp. produced Y(II) values of ~0.3-0.4 (**Figures 1A-C**). Limnic *P. wetzelii* (D300_025) showed intermediate Y(II) values (**Figure 1E**). We then followed the Y(II) for 2.5 months, showing that differences between strains persisted. The two limnic strains showed the highest Y(II) (**Figures 1D, E**), while values of *N. criophiliforma*, *C. gerlachei* and *Melosira* sp. remained lower throughout the 2.5 months (0.3-0.4) (**Figures 1A-C**). The Y(II) kinetics displayed overall constant Y(II) values in most strains (**Figure 1**); only *C. gerlachei* showed a statistically significant Y(II) decrease to ~70% after 2.5 months when compared to the values measured on day one (**Figure 1B**). The Y(II) of other strains either showed a minor increase (*N. criophiliforma*, *Melosira* sp.) (**Figures 1A, C**) or decrease (*P. wetzelii* (D300_015 + 25)) (**Figures 1D–F**) over the 2.5 months of light exposure. We next measured the Y(II) after 4 and 5 months of constant light adaptation and found Y(II) values >0.5 in all five strains (**Figure 1F**), suggesting that the photosynthetic apparatus required several months to recover. Y(II) measurements estimate indirectly, via chlorophyll fluorescence properties, how efficiently light is utilized for photochemical processes but provide little information about direct photosynthetic parameters, such as O_2_ production rates. Even though the Y(II) values at the beginning and end of the light adaptation phase of 2.5 months did not differ significantly in most strains (**Figure 1**), it can be assumed that the assimilation performance of light-adapted variants is significantly higher than that of dark-adapted ones, for example, due to the recovery of the reduced chloroplast ultrastructure under light (see below).

**Figure 3:**
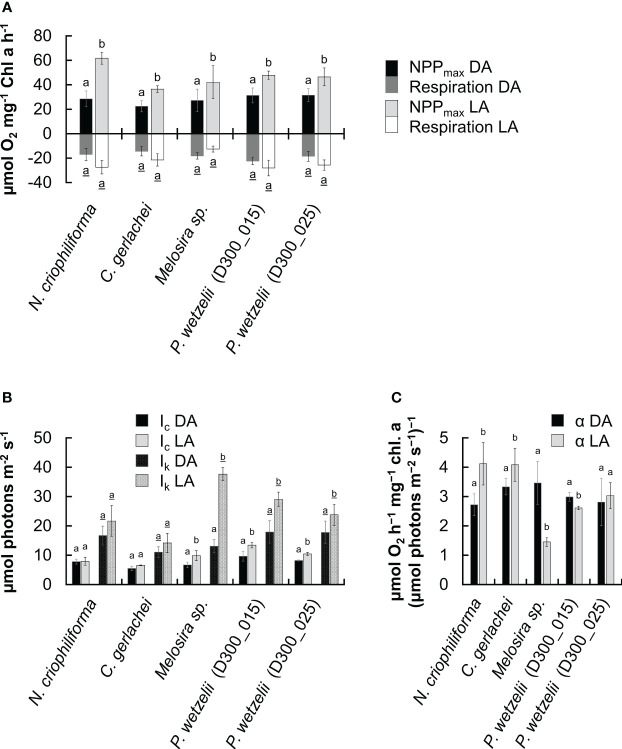
Comparison of five photosynthetic parameters derived from fitted PI curves (Walsby, 1997) in five Antarctic benthic diatoms (n=4 ± SD). Dark-adapted (10 months; DA) and light-adapted (2.5 months; LA) strains were compared. **(A)** Maximum net primary production (NPP_max_) and respiration. **(B)** Light compensation (Ic) and Ik point, expressing the initial value of light-saturated photosynthesis. **(C)** α value, PI curve slope at limiting PPFD. Significantly different means between DA and LA groups within each strain are indicated by small letters (underlined for respiration values in **(A)**). Comparison was performed by a student’s t test (p<0.05) or one-way ANOVA followed by Tukey’s *post hoc* test (p<0.05).

We therefore compared the photosynthetic oxygen production and respiratory consumption in response to increasing photon fluence rates (PI curves) between dark adapted (10 months) and light-adapted cells (2.5 months) (**Figures 2A-E**). In most strains, the maximum net primary production (NPP_max_) of dark-adapted strains was significantly lower than in light-adapted strains, but never fell below ~60% of the light-adapted cultures (**Figure 3A**). Differences in respiration between dark- and light-adapted strains were less pronounced; while most strains tended to show lower respiratory O_2_ consumption in the dark, this trend was not statistically significant (**Figure 3A**). PI curves suggested that light-adapted strains lacked strong photoinhibition, even at high photon fluence rates >1500 µmol photons m^-2^ s^-1^, while O_2_ production of dark-adapted *N. criophiliforma*, *C. gerlachei* and *Melosira* sp. (**Figures 2A-C**, **3A**) dropped to 20-30% of their NPP_max_ values at high light. Dark-adapted limnic strains showed less photoinhibition than their marine counterparts. In all strains, both the light compensation point (I_c_) and the I_k_ point, the ladder expressing the initial value of light-saturated photosynthesis, were higher in light-adapted strains, which was statistically significant in *Melosira* sp. and *P. wetzelii* (D300_015 + 025) (**Figure 3B**). The α value, which describes the PI curve slope at limiting photon fluence rates, differed among dark and light adapted strains, with the exception of *P. wetzelii* (D300_015) (**Figure 3C**). Overall, these PI curve-derived values suggest a low-light adaptation for all strains tested, indicated by low I_c_ and I_k_ values and high α values.

### 3.2 Membrane integrity, algal growth, and ultrastructure

**Figure 4:**
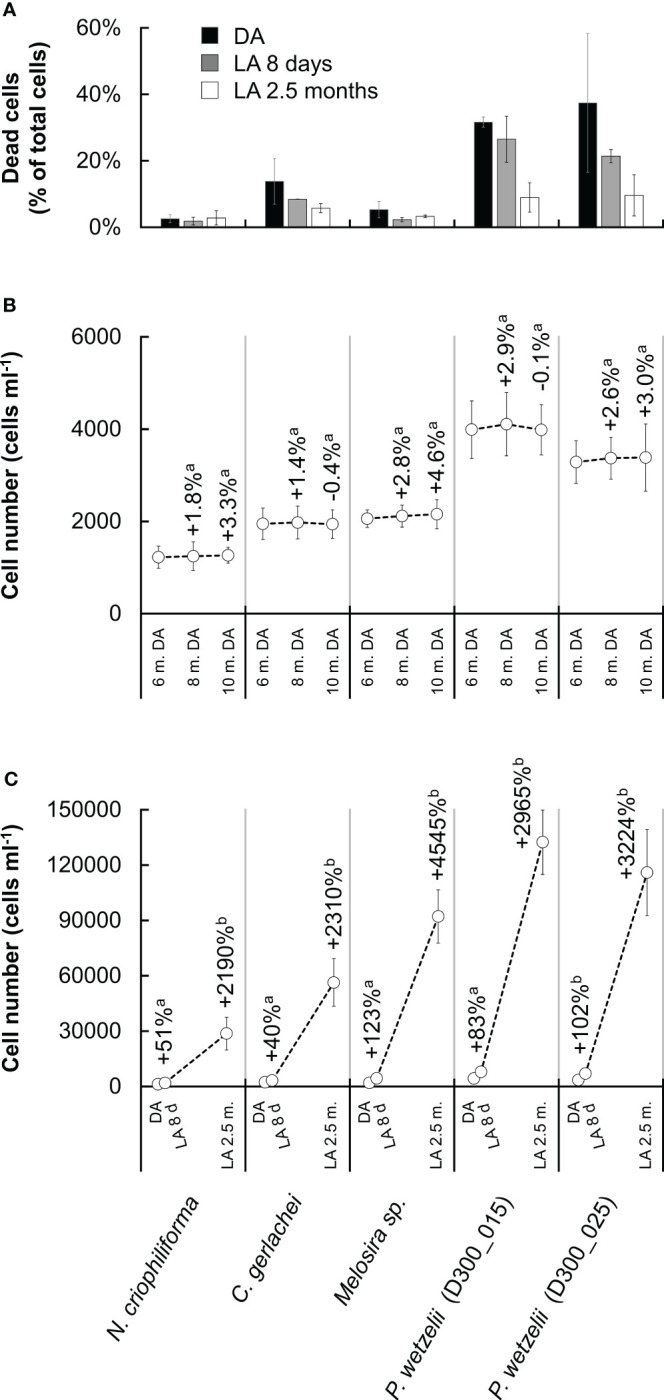
Membrane integrity and growth rates in five Antarctic benthic diatoms. **(A)** Cells stained with SYTOX Green (i.e. compromised membranes) as percentage of total cells counted after dark adaptation for 10 months (DA) and after light adaption (15 µmol photons m^-2^ s^-1^) for 8 days (LA 8 days) and 2.5 months (LA 2.5 months). At least 400 cells were counted per sample (n=2 ± SD). **(B)** Cell number in cultures during dark adaptation, counted after 6, 8 and 10 months. **(C)** Cell number in cultures after dark adaptation for 10 months (DA) and after light adaption (15 µmol photons m^-2^ s^-1^) for 8 days (LA 8 d) and 2.5 months (LA 2.5 m.). The increase of cells during light exposure is shown as a percentage above data points n=6 ± SD).

To assess whether membrane integrity contributed to long-term dark survival, Sytox Green staining was applied. The percentage of cells with compromised membranes differed strongly between strains and amounted to ~30-40% in the two limnic strains (*P. wetzelii* (D300_015 + 25)) but was much lower in the three marine strains (2~15%) (**Figure 4A**). When exposing cultures to light for 8 days, the percentage of compromised cells decreased, and a further decrease was found in strains adapted to light for 2.5 months, where the number of compromised cells was <10% in all strains (**Figure 4A**).

Counting cells after 6, 8 and 10 months of dark exposure indicated a slight increase by up to ~4% from month 6 to month 10 (**Figure 4B**), however, this was not statistically significant (p<0.05). The cell numbers after dark exposure differed strongly among treatments, ranging from 1200 to 4300 cells ml^–1^ (**Figure 4C**). Light exposure for 8 days increased the cell number significantly (p<0.05) in all strains, up to doubling the number in cultures of marine *Melosira* sp. and in both limnic *P. wetzelii* cultures. Light exposure for 2.5 months produced cell numbers of >130.000 cells in *P. wetzelii* (D300_015) cultures (**Figure 4C**).

**Figure 5:**
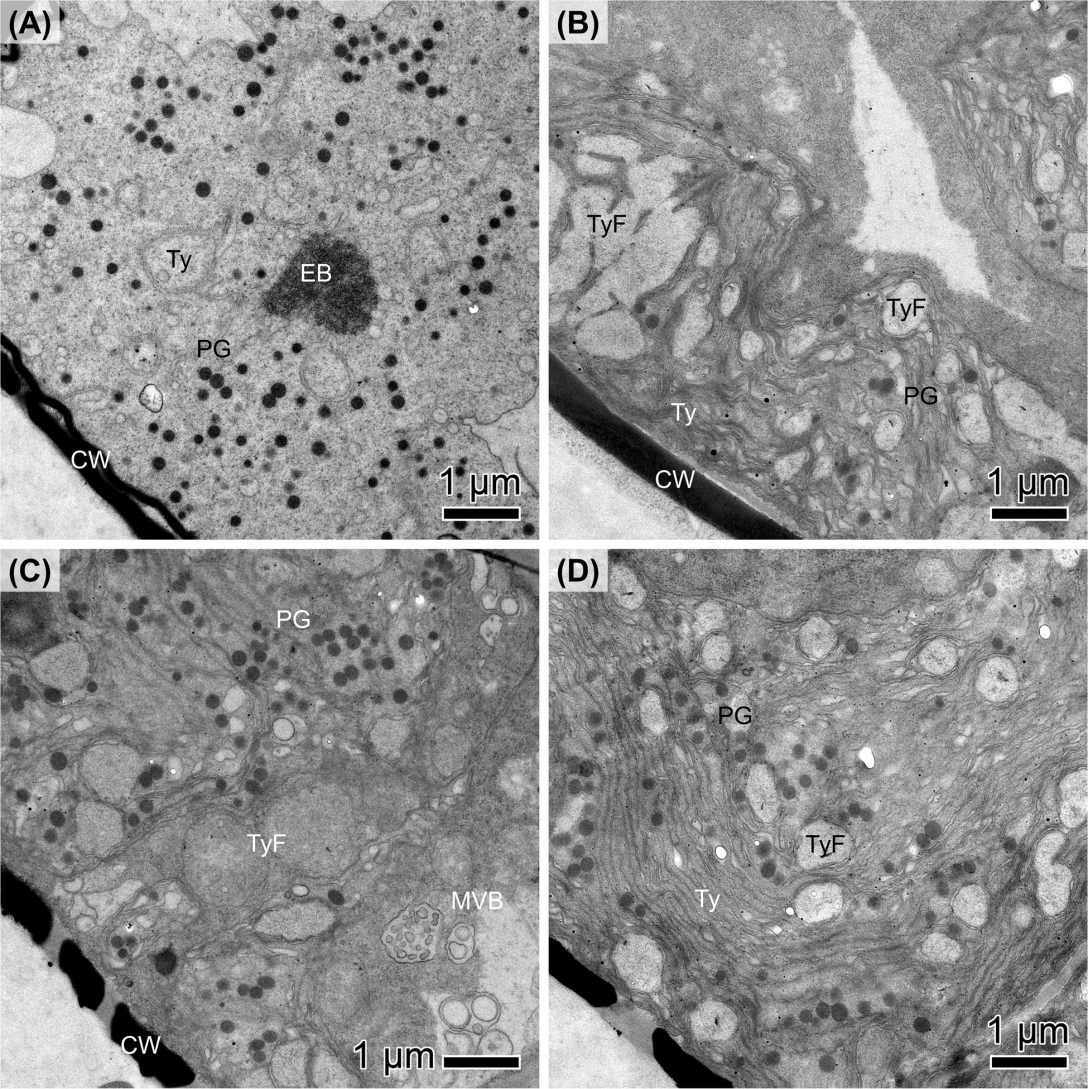
Transmission electron micrographs of **(A, B)** *Navicula criophiliforma* and **(C, D)** *Planothidium wetzelii* (D300_015) after **(A, C)** 10 months of dark exposure, followed by **(B, D)** 2.5 months at light. **(A)** Chloroplast with disorganized thylakoid (Ty) membranes, numerous plastoglobulus (PG) and electron-dense structures (EB); the cell wall (CW) is adjacent to the chloroplast. **(B)** Chloroplast with few plastoglobules, clearly visible thylakoid stacks and thylakoid-free spaces (TyF). **(C)** Chloroplast with numerous plastoglobules, disorganized thylakoids and multivesicular bodies (MVB); the cell walls exhibit pores. **(D)** Numerous plastoglobules but with thylakoids organized in stacks. Scale bars: 1 μm.

TEM was used to examine the cellular ultrastructure of 10 months dark-adapted diatoms and compared to cells adapted to light for 2.5 months after dark exposure. Here, a marine (*N. criophiliforma*) and limnic (*P. wetzelii* (D300_015)) strain is exemplified. Both strains exhibited an appreciable Y(II) after dark exposure (**Figures 1A, D**), even though both dark-adapted strains possessed a significantly reduced and less organized chloroplast ultrastructure (**Figures 5A, C**). The thylakoids were scattered, hardly organized into grana, and numerous plastoglobules were visible (**Figures 5A, C**). Moreover, multivesicular bodies (MVBs) were found in *P. wetzelii* (D300_015) (**Figure 5C**). In light-adapted strains, the chloroplast ultrastructure appeared regenerated as evident by pronounced thylakoid stacks (grana), especially in *P. wetzelii* (D300_015) (**Figure 5D**), indicating a good structural condition of the chloroplasts. This is well in line with the improved photosynthetic performance in light-adapted strains (**Figures 2A, E**). Moreover, a significantly lower plastoglobule density was evident in light-adapted strains. *P. wetzelii* (D300_015) contained multivesicular bodies and clearly identifiable cell wall pores (**Figure 5C**).

**Figure 6:**
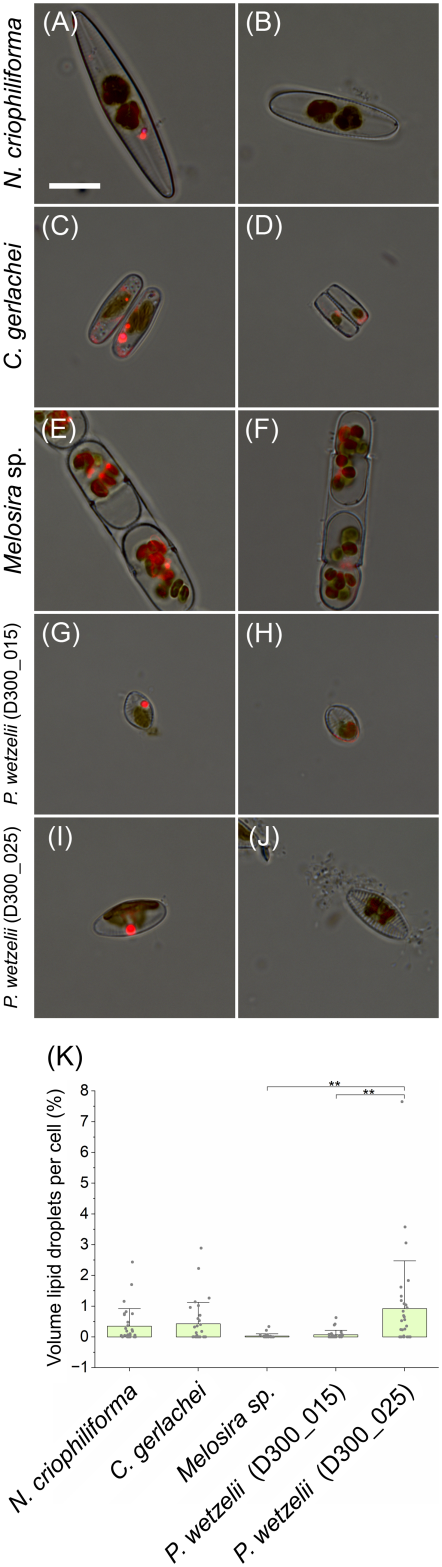
Quantification of cellular lipid deposition. Lipid droplets are shown as bright red fluorescence signal and these images were merged with bright field images. **(A, C, E, G, I)** Cells containing lipid droplets, **(B, D, F, H, J)** cells without lipid droplets. **(K)** Amount of lipid droplets in cells (in % of cell volume), averaging between 0 and 1% in *N. criophiliforma*, *C. gerlachei* and *P. wetzelii* (D300_025). *Melosira* sp. and *P. wetzelii* (D300_015) showed the highest proportion of cells with complete lipid droplet depletion. Significant difference between means (n=30 ± SD) were determined via one-way ANOVA (p<0.01, **). Scale bar = 10 µm.

Quantifying the total lipid content detectable by nile red after 10 months of dark incubation revealed that cells only contained few small lipid droplets (**Figure 6**). Overall, <1% of the cell volume was occupied by lipid droplets (**Figures 6A, C, E, G, I, K**) and many cells showed complete lipid depletion (**Figures 6B, D, F, H, J**).

## 4 Discussion

Here we showed that both limnic and marine Antarctic diatoms from benthic habitats sustain prolonged dark exposure for 10 months and show an appreciable photosynthetic performance as soon as light becomes available. This might be key for their survival as phototrophic organisms in polar regions, where they face extreme light fluctuations. A recent review (Pavlov et al., 2019) summarized the complex underwater light conditions in the well-studied Kongsfjorden in the Arctic, which is used here as a representative example due to limited Antarctic data. Factors like seasonal changes, clouds, sea ice, and water properties affect light penetration. Run-off, glacial meltwater, and phytoplankton blooms add complexity. Similar conditions likely exist in Potter Cove and other Antarctic Peninsula bays (Hoffmann et al., 2019). Additionally, glacier retreat and global change introduce more particles, reducing available light (Hoffmann et al., 2019). Thus, studying the photophysiological responses of diatoms to extreme light conditions is timely and crucial to understand their reactions to changing environments.

### 4.1 Dark exposure has long-lasting effects on photosynthesis

A recent study assessed the photosynthetic performance of the same diatom strains investigated in the present work and found Y(II) values of ~0.6 for light-adapted strains (Juchem et al., 2023). Earlier studies on other Antarctic benthic diatoms reported similar values (0.6-0.7; Longhi et al., 2003; Wulff et al., 2008). While Juchem et al. (2023) found no decrease of Y(II) after 3 months of darkness, we showed that 10 months of dark exposure reduced these values in all strains and no strain exceeded a Y(II) of >0.6, suggesting that prolonged lack of light impairs the physiological condition of diatoms. Moreover, a reduced Y(II) remained for at least 2.5 months at light after 10 months of darkness. Recovery was only possible after 4-5 months of cultivation under light, restoring Y(II) values to >0.5 in all five strains (**Figure 1F**). Interestingly, the photosynthetic oxygen production did not fully reflect Y(II) measurements. In all investigated strains, NPP_max_ values where relatively low after 10 months of darkness, however, a strong increase of O_2_ production was measurable after 2.5 months of light exposure. In contrast, the Y(II) hardly changed during these 2.5 months of light exposure or even declined in some strains. It possible that the recovery after dark exposure strongly improves the carboxylation capacity, while the light harvesting efficiency – measured as Y(II) – increases to a lesser extent or requires more than 2.5 months to recover. This is suggested by PI curve parameters, where in all 5 strains, NPP_max_ increases >1.5-fold during recovery under light for 2.5 months (**Figure 3A**); in contrast, the α value, which reflects the photosynthetic efficiency at the light-limiting range, increases <1.5-fold or even declines in some strains (**Figure 3C**).

Decreasing NPP_max_ in Antarctic benthic diatoms after ~2 months of dark exposure was found before and explained by a degradation of antennae complexes in the photosynthetic reaction centers (Wulff et al., 2008), which likely coincides with a decline in chlorophyll *a* concentration (Juchem et al., 2023) and reduction of light-harvesting complexes and photosystems (Kennedy et al., 2019). Correspondingly, in all five strains, we found a significantly lower chlorophyll *a* content in dark adapted cells when compared with cells exposed to light for up to 2.5 months (**Supplementary Figure S2**). *N. criophiliforma* (~7 ng chlorophyll *a* per cell; **Supplementary Figure S2**), *Melosira* sp. (~4.5 ng chlorophyll *a* per cell; **Supplementary Figure S2**) and both *P. wetzelii* strains (D300_015 + 25; 3.3 and 4.1 ng chlorophyll *a* per cell) had similar chlorophyll *a* contents per cell after 2.5 months light exposure following a 10 months dark period as reported by Juchem et al. (2023) for cells from light-adapted cultures. In contrast to Juchem et al. (2023), we found a ~7 times higher chlorophyll *a* content per cell in *C. gerlachei* (~3 ng chlorophyll *a* per cell). This content is well within the range of values measured in the other four strains (see **Supplementary Figure S2** and Juchem et al., 2023). Moreover, considering that *C. gerlachei* showed an overall similar photophysiological behavior to the four other strains (e.g., **Figure 3**) and all five strains were isolated from similar habitats, similar chlorophyll *a* contents can be expected. Thus, we suggest that the differences in *C. gerlachei*’s chlorophyll *a* content between the present study and Juchem et al. (2023) might be due to an underestimation in the earlier study. Importantly, a drop in chlorophyll *a* content during dark adaption for up to 10 months was consistently found in all strains tested (**Supplementary Figure S2**). While the chlorophyll *a* content strongly decreased during long-term dark adaption, sampling in months 6-10 of the 10 months dark exposure period indicated a minor trend for increasing cell numbers (3-5% from month 8 to 10), which, however, was not statistically significant.

### 4.2 Prominent changes of chloroplast ultrastructure after dark and light adaption

The chloroplast ultrastructure showed clear signs of degradation, most prominently a strongly reduced and disorganized thylakoid structure and high abundance of plastoglobules. These are osmophilic lipid bodies that often occur in association with stressed chloroplasts (van Wijk and Kessler, 2017). Plastoglobules contain thylakoid building blocks and several proteins that are involved in lipid metabolism or the structural integrity of plastoglobules, such as fibrillins, which might help stabilizing the globules and preventing their coalescence (Rottet et al., 2015). As found for land plants, both darkness or continuous light for 7 days can down- and upregulate certain fibrillins, suggesting that the formation dynamics and maintenance of plastoglobules is strongly linked to the light conditions (Ytterberg et al., 2006). As plastoglobules appear to actively participate in thylakoid biogenesis to senescence, it is possible that Antarctic diatoms “store” most of their thylakoids as plastoglobules during long-term darkness; as soon as light becomes available, they can be used as building blocks to restore the grana system, allowing to regain an appreciable photosynthetic performance. Degradation of the chloroplast seems to be a key mechanism in benthic diatoms to survive the polar night; dark exposure condenses the chloroplast in Antarctic diatoms (Wulff et al., 2008), and reduces the chloroplast lengths by up to 50% in Arctic diatoms (Karsten et al., 2012, 2019b), but recovery is possible after some hours in light. Even though strongly reduced, after 10 months of darkness, thylakoids are still visible in plastoglobules-rich chloroplasts, helping to explain why appreciable photosynthetic oxygen production is measurable immediately upon light exposure.

Another possibility is that chloroplast lipids are converted to plastoglobules and ultimately transferred to the cytoplasm, degraded by autophagy to mobilize energy reserves and allow cells to remain metabolically active during prolonged darkness. Such mechanism was found in green algal model system *Micrasterias* (Schwarz et al., 2017). However, cells adapted to darkness for 10 months showed a high abundance of plastoglobules (**Figure 5**), suggesting that plastoglobules do not serve as a major energy resource. Measurable photosynthesis in diatoms investigated in the present study suggest that none of them relied on metabolically inactive resting cells to survive prolonged darkness. Similar results were found for the Antarctic diatom *Thalassiosira tumidu*, which survived for 9 months in darkness in the vegetative state and regained an appreciable growth rate when exposed to light again (Peters and Thomas, 1996). Considering the dynamic degradation processes in chloroplasts during darkness, it is remarkable that diatoms can maintain an appreciable photosynthetic capacity in the absence of light for 10 months. On the other hand, full recovery of the photosynthesis proxy Y(II) took several months, suggesting that diatom populations facing long-term dark exposure can never regain their full photosynthetic potential after darkness and when exposed to relatively low light (15 µmol photons m^-2^ s^-1^) for less than 4-5 months. Nevertheless, all strains produced appreciable cell numbers during 2.5 months under such light conditions (see. **Figure 4C**), which might be explained by their low light requirements as PI curve parameters suggest; however, it is possible that higher irradiances (e.g. 50 µmol photons m^-2^ s^-1^; Peters and Thomas, 1996) would allow for a quicker recovery of the Y(II).

Long-term dark survival can be enhanced significantly by the formation of resting cells (cysts; McMinn and Martin, 2013), allowing cells to survive with minimal effort on cellular energy. When buried in benthic sediments, such cells enable *Melosira* sp. to survive for 20 years (Sicko-Goad et al., 1986) and certain other marine diatom genera (e.g., *Chaetoceros*, *Skeletonema*) can even survive for up to 100 years as resting cells and germinate as soon as brought back to light (Lundholm et al., 2011). The latter taxa exhibit a planktonic life-style, and Smetacek (1985) discussed the mass sinking of such diatom cells after a bloom event as a transition from a growing phase to a resting stage in the life history of these protists. Mass sinking into the dark region of the water column is considered as ecologically important since these diatom cells often retain viability under cold conditions, which is particularly true in coastal regions (Smetacek, 1985). Such deep-water resting stages are considered as seeding population when freshly recycled nutrients are available (Smetacek, 1985). This makes diatom resting cells a potential source of phytoplankton to their overlying waters as soon as disturbance releases them from dormant state and exposes them to light (Lewis et al., 1999).

### 4.3 Membrane integrity differs between marine and limnic strains

Few studies investigated the cell and membrane integrity of polar benthic diatoms. In a dark exposure experiment, Arctic *Surirella* cf. *minuta* cultures exhibited ~20% dead cells after 1 month incubation (Karsten et al., 2019a). In contrast, after 5 months of dark exposure, >95% of Arctic *Navicula directa* cells were still alive as indicated by their intact membrane systems (Karsten et al., 2019b). This suggests that some diatom cells can cope better with long-term darkness than others, which we also confirmed after 10 months of dark-exposure of Antarctic strains. Interestingly, both limnic strains [*P. wetzelii* (D300_015 + 25)] exhibited a higher proportion of cells with compromised membranes (~1/3) than their marine counterparts, in some of which ~95% of cells were not compromised (**Figure 4A**). However, dark exposure for 3 months led to a higher proportion of compromised cells in these strains, spanning ~20-60% (Juchem et al., 2023). It is possible that these marine diatoms undergo a lengthy adaption process to darkness, allowing them to establish a higher membrane integrity over time. In contrast, the limnic strains appear to have a lower cell lifespan (more cells with compromised membranes) yet higher cell proliferation rate as indicated by higher growth rates after a dark period (**Figure 4C**). This might suggest different strategies to maintain appreciable cell numbers during dark and light periods, i.e. improving membrane integrity during darkness or allowing for higher proliferation rates in the light. When transferred to light after 10 months of darkness, the cell number recovered relatively slowly during a period of 8 days and was higher after 2.5 months, suggesting that cell division processes require time to recover under light. Overall, these observations are in line with earlier studies suggesting that diatoms cultured in darkness for several months do not grow; however, cells entered proliferation immediately after light exposure (Miquel, 1892). Lewin (1953) found that in the presence of glucose, several *Navicula* spp. show “unlimited” growth in the dark, at least for 2 months. While these studies added carbon sources to culture media to support heterotrophic growth, we did not supplement glucose or other compounds for heterotrophic growth, also preventing bacterial growth in non-axenic cultures. This suggests that the 5 Antarctic diatom strains tested can sustain cellular survival in the vegetative state, while showing relatively high respiration as indicated by a respiration rate of ~15-20 µmol O_2_ mg^-1^ chlorophyll *a* h^-1^ (**Figure 3A**), which was measured immediately after the 10 months dark-period. Assuming a respiratory quotient of 0.8 (Lewin et al., 1955) and constant respiration rates, this would result in a respiratory CO_2_ production of up to ~16 µmol CO_2_ mg^-1^ chlorophyll a h^-1^ and thus a carbon loss of ~192 µg C mg^-1^ chlorophyll a h^-1^, resulting in a total carbon loss of ~1400 C mg^-1^ chlorophyll *a* in 10 months. Such carbon loss might be even higher, because diatom cultures exposed to darkness for 3 months showed a higher respiratory O_2_ consumption (up to ~36 µmol O_2_ consumption per mg chlorophyll *a* and hour; Juchem et al., 2023). In part, this might be explained by co-occurring heterotrophic bacteria in cultures, however, we consider this risk relatively low as removing diatom cells from cultures showed low O_2_ consumption, assuming that co-occurring bacteria do not strongly stick to diatoms and remained in cultures. *P. wetzelii* (D300_015 + 025) showed relatively high respiration rates, which coincided with a high proportion of dead cells in cultures (~33%; **Figure 4A**) and cells might have used organic matter released from dead cells as a substrate to sustain a relatively high heterotrophic metabolism. In general, high respiration levels during prolonged darkness of polar night are not uncommon (Berge et al., 2015) and our data confirm that this can also be true for Antarctic diatoms. Antarctic diatoms can use their lipid storage pools during long-term darkness. During 3 months of darkness, marine *N. criophiliforma*, *C. gerlachei*, and *Melosira* sp. used up ~90% of their lipids, while the content decreased by less than 40% in limnic *P. wetzelii* (D300_015 + 025) (Juchem et al., 2023). As confirmed by nile red staining, the cellular lipid pool is depleted after 10 months of dark inception, where only <1% of the cellular volume remained occupied by lipid droplets in all 5 strains. Drawing energy from the lipid pool via autophagy or lipophagy during darkness was also suggested for the polar diatom *Fragilariopsis cylindrus* (Joli et al., 2023). Another source for energy in benthic diatoms can be anaerobic respiration using intercellular storage pools of NO_3_^-^ to reduce them to NH_4_ (Kamp et al., 2011). However, depletion of NO_3_^-^ occurs very rapidly, suggesting that the energy provided by dissimilatory NO3- reduction might be sufficient for entering a resting stage but cannot directly fuel long-term dark survival (Kamp et al., 2011).

In conclusion, we found that long-term dark-exposure strongly reduced the photosynthetic performance and chloroplast ultrastructure in all five Antarctic benthic diatoms. Nevertheless, algae showed appreciable photosynthesis rates as soon as darkness ceased, while full recovery of photosynthesis required several months under light. Future studies may explore the molecular mechanism leading to the observed long-term down-regulation of photosynthesis. In an ecological context, it will be interesting to explore whether prolonged darkness of several months, for example due to polar night and ongoing ice coverage, followed by a short light periods (2-4 months) and another dark period, such as the next polar night, would allow for long-term population survival or whether such light periods are too short to fully restore photosynthesis, accumulate sufficient assimilates, and sustain the next period(s) of darkness.

## Data availability statement

The original contributions presented in the study are included in the article/**Supplementary Material**. Further inquiries can be directed to the corresponding author.

## Author contributions

JH: Writing – review & editing, Writing – original draft, Visualization, Validation, Investigation, Formal analysis, Conceptualization. DJ: Resources, Writing – review & editing, Validation. QW: Visualization, Investigation, Formal analysis, Writing – review & editing, Validation. KS: Writing – review & editing, Validation, Resources, Investigation. OS: Writing – review & editing, Validation, Resources, Investigation. JZ: Writing – review & editing, Validation, Resources, Funding acquisition. UK: Writing – review & editing, Validation, Resources, Project administration, Methodology, Funding acquisition, Conceptualization. KH: Writing – review & editing, Writing – original draft, Visualization, Validation, Supervision, Resources, Project administration, Methodology, Investigation, Funding acquisition, Formal analysis, Data curation, Conceptualization.

## Supplementary material

Supplementary Figure 1 Isolation sites of diatoms in Potter Cove. **(A)** Map of Antarctica. **(B)** Overview King George Island). **(C)** Potter cove with 3 marine (APC06, APC14, APC41) and 1 limnic (APC18) diatom isolation sites. Maps were obtained via Google Maps.

Supplementary Figure 2 Chlorophyll content in 5 Antarctic diatom strains after 6 and 10 months of dark adaptation (DA) and 8 days and 2.5 months of light adaption (LA). Comparison of mean values was done using one-way ANOVA followed by Tukey’s *post hoc* test (p<0.05) and different small letters indicate significantly different values for each diatom strain.

Supplementary Table 1 List of diatom samples collected at Potter Cove, King George Island (Antarctica) with information about the 5 strains and the sampling site.
