# Supplementary figures and images for: Antarctic benthic diatoms after 10 months of dark exposure: consequences for photosynthesis and cellular integrity
Source: Front Plant Sci. 2024 Mar 22;15:1326375. doi: 10.3389/fpls.2024.1326375 (PMC10995292; doi:10.3389/fpls.2024.1326375)

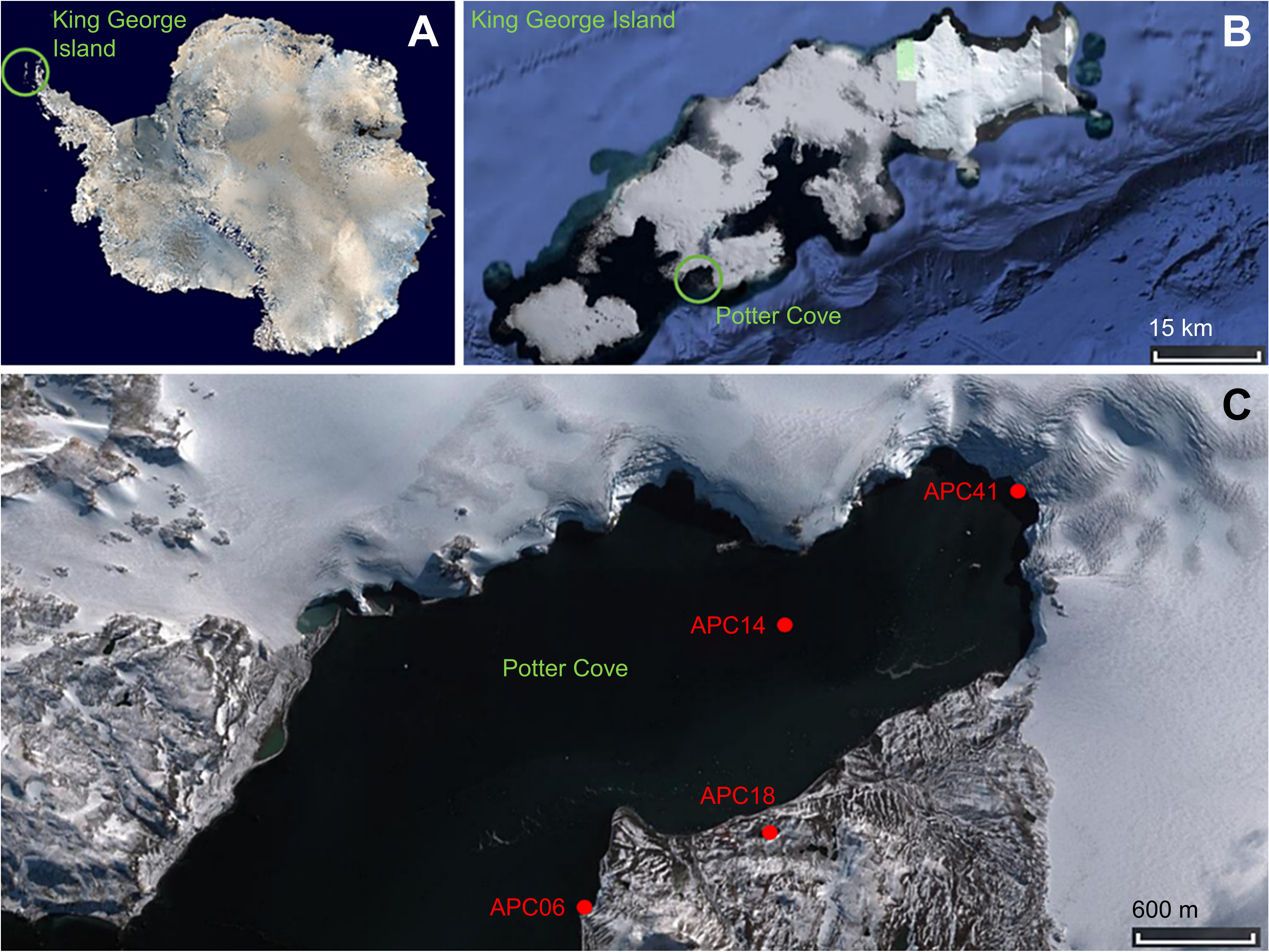

Supplement: Supplementary Figure 1 — Isolation sites of diatoms in Potter Cove. (A) Map of Antarctica. (B) Overview King George Island). (C) Potter cove with 3 marine (APC06, APC14, APC41) and 1 limnic (APC18) diatom isolation sites. Maps were obtained via Google Maps. [file Image_1.tif]

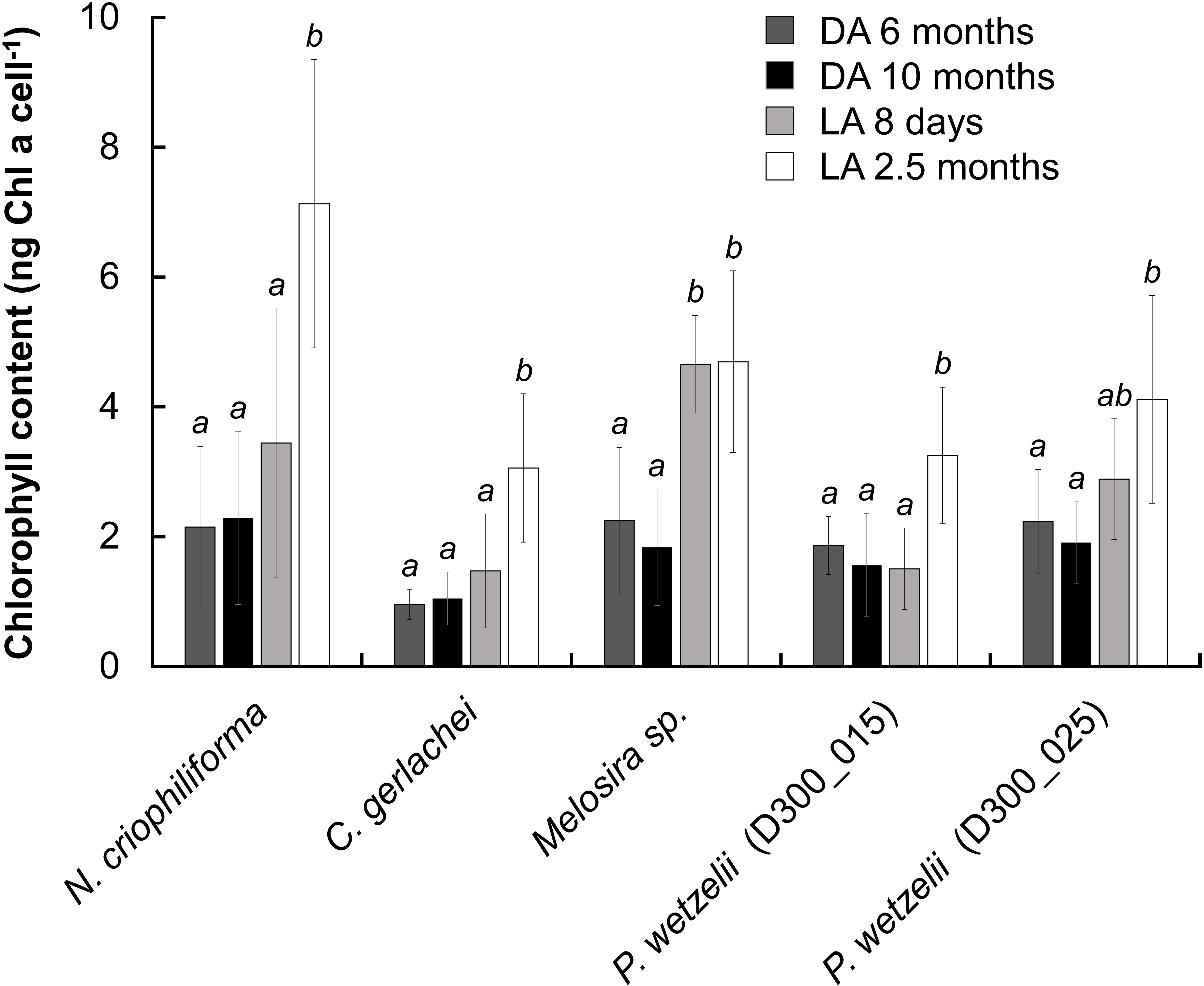

Supplement: Supplementary Figure 2 — Chlorophyll content in 5 Antarctic diatom strains after 6 and 10 months of dark adaptation (DA) and 8 days and 2.5 months of light adaption (LA). Comparison of mean values was done using one-way ANOVA followed by Tukey’s post hoc test (p<0.05) and different small letters indicate significantly different values for each diatom strain. [file Image_2.tif]
