# Supplementary material for: Antarctic benthic diatoms after 10 months of dark exposure: consequences for photosynthesis and cellular integrity
Source: Front Plant Sci. 2024 Mar 22;15:1326375. doi: 10.3389/fpls.2024.1326375 (PMC10995292; doi:10.3389/fpls.2024.1326375)
Supplement: Supplementary Table 1 — List of diatom samples collected at Potter Cove, King George Island (Antarctica) with information about the 5 strains and the sampling site. [file Table_1.pdf]

Isolation site	Coordinates	Habitat, sampling information	Water type	Species and strain no.	Lengths (µm)	Width (µm)
APC06	S 62°14'30.55", W 58°40'54.96"	biofilm, intertidal rock pool, rock surface, end of low tide	marine	*Navicula criophiliforma* D288_003	34.4 – 44.9	5.1 – 8.5
APC14	S 62°13'43.61", W 58°39'49.36"	episammic biofilm, upper layer sediment taken with syringe, 3 cores pooled	marine	*Chamaepinnularia gerlachei* D296_001	7.2 – 15.4	2.2 – 5.6
APC41	S 62°13'25.68", W 58°38'33.50"	episammic biofilm, upper layer sediment taken with syringe, 3 cores pooled	marine	*Melosira* sp. D323_018	11.5 – 28.8 (valvar diameter)	6.7 – 10.1 (pervalvar axis)
APC18	S 62°14'16.30", W 58°39'44.10"	Biofilm on 5 stones	limnic	*Planothidium wetzelii* D300_015	4.3 – 9.8	2.4 – 6.1
APC18	(same as line before)	Biofilm on 5 stones	limnic	*Planothidium wetzelii* D300_025	11.9 – 17.9	3.6 – 6.6
